# Selections and Abstracts

**Published:** 1915-09

**Authors:** 


### SELECTIONS AND ABSTRACTS

### EXAMINATION OF CANDIDATES FOR ASSIST SURGEON.—TREASURY DEPARTMENT.

United States Public Health Service
Washington, Sept. 7, 1915.

Boards will be convened at the Bureau of Public Health Service, 3 "B" Street, S. E., Washington, D. C., and at the Marine Hospitals of Boston, Mass., New York, N. Y., Chicago, Ill., St. Louis, Mo., Louisville, Ky., New Orleans, La., and San Francisco, Cal., on Monday, November 1, 1915, at 10 o'clock a. m., for the purpose of examining candidates for admission to the grade of Assistant Surgeon in the Public Health Service.

Candidates must be between 23 and 32 years of age, graduates of a reputable medical college, and must furnish testimonials from two responsible persons as to their professional and moral character. Credit will be given in the examination for service in hospitals for the insane or experience in the detection of mental diseases. Candidates must have had one year's hospital experience or two years' professional work.

Candidates must be not less than 5 feet, 4 inches, nor more than 6 feet, 2 inches, in height, with relatively corresponding weights.

The following is the order of examination: 1, Physical; 2, Oral; 3, Written; 4, Clinical.

Candidates are required to certify that they believe themselves free from any ailment which would disqualify them for service in any cilmate.

Examinations are chiefly in writing, and begin with a short autobiography of the candidate. The remainder of the written exercise covers the various branches of medicine, surgery, and hygiene.

The oral examination includes subjects of preliminary education, history, literature, and natural sciences.

The clinical examination is conducted at a hospital.

The examination usually covers a period of about ten days.

Successful candidates will be numbered according to their attainments on examination, and will be commissioned in the same order. They will receive early appointments.

After four years' service, assistant surgeons are entitled to examination for promotion to the grade of passed assistant surgeon. Passed Assistant Surgeons after twelve years' service are entitled to examination for promotion to the grade of Surgeon.

Assistant Surgeons receive $2,000, passed assistant surgeons $2,400, surgeons $3,000, senior surgeons $3,500, and assistant surgeon-generals $4,000 a year. When quarters are not provided, commutation at the rate of $30, $40, and $50 a month, according to the grade, is allowed.

All grades receive longevity pay, 10 per cent. in addition to the regular salary for every five years up to 40 per cent. after twenty years' service.

The tenure of office is permanent. Officers traveling under orders are allowed actual expenses.

For invitation to appear before the board of examiners, address "Surgeon-General, Public Health Service, Washington, D. C."

### CONTROLLING CANCER IN ENGLAND.

Portsmouth was the first municipality in England to undertake a public educational campaign for the control of cancer and it would appear that the measures adopted in 1913 are already taking effect. The annual report of the Medical Officer of Health, Dr. A. Mearns Fraser, for the year 1914, which has just been received, states that there were only 197 deaths from cancer in Portsmouth last year as compared with 230 in 1913. This decrease, which occurs in the face of an increase in population, is hailed with satisfaction by the Portsmouth sanitary authorities as justifying their efforts to reduce the cancer death rate by persuading persons who are attacked with this disease to avoid delay and to seek treatment before it is too late for more than palliative measures. Dr. Fraser reports that from statements made to him by local medical men the publication of circulars and newspaper articles by the Health Department has been instrumental in inducing a number of persons suffering from early operable cancer to secure treatment, the result of which it is hoped will be permanent.

When the educational measures were put in force two years ago, the cancer death rate of the city had for a long period been increasing. Twenty years ago the average death rate from cancer in Portsmouth was 6.79 per 10,000 of the population, but in 1913 it had risen to 9.16 per 10,000. In that year the total number of deaths was only 34 less than were caused by tuberculosis. While admitting that the increase in the recorded cancer death rate might have been caused in part by improved methods of diagnosis, the Health Committee of the Portsmouth Town Council nevertheless believed that the present number of deaths was unnecessarily large, and they felt it incumbent to adopt whatever measures might lessen the ravages of the disease. The initiative came from Dr. Charles P. Childe, senior surgeon of the Royal Portsmouth Hospital and a member of the Health Committee of the Town Council. As early as 1906 Dr. Childe in his book "The Control of the Scourge" had given to the public the benefit of his extended experience with cancer.

At his suggestion the Portsmouth authorities in 1913 began a campaign of public education under the official auspices of the Health Department. The methods adopted included the monthly publication in the local newspapers of articles regarding cancer and the printing and distribution of a Health Department circular on the subject. Arrangements were made for periodical lectures to midwives, nurses, and to those engaged in social work in Portsmouth. The Health Department further made provision for free microscopical examinations and reports on suspected cancerous growths in order to assist physicians in immediate diagnosis in the case of patients who were unable to pay for such laboratory service. The experience of the Portsmouth authorities had been that by far the majority of patients who presented themselves at hospitals suffering from cancer exhibited the disease in a stage too advanced to be cured. It was held that the reason for this delay in seeking advice was not as a rule because patients feared operation, but because they were ignorant that they were suffering from anything serious until they began to suffer pain. The fact that cancer at its onset is almost always painless should be widely realized in order that the public may learn the importance of other symptoms which will enable them to recognize the disease in the early stages when it can nearly always be successfully removed by competent surgery.

---

### THE CAMPAIGN AGAINST CANCER IN MISSOURI.

The most recent addition to the many agencies, national and local, now engaged in the warfare on cancer is the Department of Preventive Medicine of the University of Missouri. This Department has just published in the University Bulletin a special article on the early diagnosis and treatment of cancer by Dr. F. A. Martin, instructor in pathology. The purpose of this bulletin is to call the attention of its readers in Missouri and elsewhere to the campaign for the education of the laity which is being carried on by the American Society for the Control of

Cancer, the American Medical Association and other national and state organizations, and to give a brief general survey of the cancer problem as a phase of preventive medicine.

The knowledge and skill of surgeons in the treatment of cancer has progressed, according to the Bulletin, almost to the limits of what is possible and if the percentage of cures by this, the only method of treatment which offers reliable hope of cure, is to be increased, the patients themselves must co-operate by seeking earlier diagnosis and treatment. On examining the histories of a large number of cases it has been found that the patients whom the surgeon failed to cure were those who came to him late in the disease when the cancer had spread to such an extent that to remove all the cancer cells would have required an operation so great that in itself it would be sufficient to cause the death of the patient. On the other hand, it is found of another group of cases which sought treatment soon after the cancer was noticed that 100% were cured. To increase the percentage of cases treated early the University Bulletin urges that laymen learn the meaning of cancer and its first warnings in order that they may go to the surgeon in time when the cancer is still in the early stages and the chance for cure is high.

Among the many facts already known about cancer, perhaps the most important is that the disease nearly always begins in some form of abnormal tissue. This abnormal tissue which is often easily recognized, may have existed for only a few months or it may have been present from early childhood without causing trouble, only to change into cancer in later life. To these bits of abnormal tissue or groups of cells, has been given the name of "precancerous lesion." The Bulletin says that not all such conditions develop into true cancers, but most of them should be kept under careful observation by a competent medical advisor and removed as soon as there is real danger of malignant disease. This is the only known method of preventing, as distinguished from curing, cancer and the Missouri Bulletin described carefully the various forms of precancerous lesions which should be regarded with suspicion. Among these are pigmented moles, cracks on the lip, blisters, scabs and similar persisting abnormal conditions of the skin. Probably only a very small proportion of these conditions become cancer, but when moles, for instance, are so located that they are subject to constant irritation and when in later life they change in color and appearance and begin to grow it is time to have them promptly attended to. Moles and warts should never be treated with caustic but the whole lesion together with its so-called roots should be removed. When a burn on the tongue or lip from smoking does not heal within a few months it is a source of danger. Generally speaking, the removal of precancerous lesions is a trivial operation requiring only local anesthesia.

After true cancer has developed it is still possible to cure a large percentage of cases if the surgeon is given a fair chance while the disease is still local. All cases of cancer are local in the beginning and may remain so for a few weeks to several months. It is during this period that surgical treatment offers the possibility of practically 100% of cures. Unfortunately for the patient pain is so rare at this stage of the disease and the conditions seem so trivial that in a great number of cases the opportunity to be saved is forfeited by the delay. In cancer of the breast, for instance, the cases cured by the late operation amount to about 30%, but by an early operation, at least 80% are saved. If every women who is not nursing would go to a surgeon within 24 hours after she finds a lump in her breast, 90% of the cases of cancer of the breast would be permanently cured.

Cancer of the tongue is perhaps the most malignant and cures by the late operation are few in number. If a small ulcer appears on the tongue, consult a surgeon at once. When such an ulcer is produced by a ragged tooth, consult a dentist and then if the ulcer does not heal within a short time after the cause has been removed, it is a surgeon's task.

In almost all the common forms cancer is connected with some kind of irritation. Gall stones, for instance, should be removed since it is established that from four to fourteen per cent. of all cases are followed by cancer.

Cancer of the uterus gives early warning by a discharge of an unusual character at an unusual period and of unusual duration. The removal of the uterus is not a dangerous operation and if the disease is recognized at an early stage the life of the patient can be saved.

The Bulletin issues an emphatic warning against quacks and their bogus testimonials, pointing out that their method of deception lies mainly in the diagnosis. There are so many conditions closely resembling cancer that the average layman cannot distinguish among them, and it is behind such conditions which are not cancer and which would tend to heal without treatment that the "cancer specialists" take their stand and make their false claims.

The Department of Preventive Medicine will supply copies of this cancer bulletin, Medical Series No. 9, upon request to the University of Missouri, Columbia, Mo., as long as the supply lasts.

---

### THE ROCKEFELLER FOUNDATION ANNUAL REPORT—PART ONE.

61 Broadway, New York, Sept. 23, 1915.

The Rockefeller Foundation will shortly issue its first annual report, covering the period to the end of 1914.

It is the plan of the Trustees to issue hereafter a full report soon after the close of each calendar year.

Problems of organization, and details in connection with reporting upon the work of the International Health Commission (which report in full is to be included in the report of the Foundation), has occasioned the delay in the issuance of the first report.

The first part of the annual report of the Rockefeller Foundation to be made public deals with the activities of the International Health Commission.

### International Health Commission

The first meetings of the Rockefeller Foundation, after its legal organization had been completed, were devoted to the discussion of the policies and lines of work which were likely to present the largest probability of permanent and far-reaching usefulness. There was a general agreement that the advancement of public health through medical research and education, including the demonstration of known methods of treating and preventing disease, afforded the surest prospect of such usefulness. It was accordingly, decided at the meeting of June 27, 1913, to establish the International Health Commission.

Mr. Wickliffe Rose, Administrative Secretary of the Rockefeller Sanitary Commission for the Eradication of Hookworm Disease, was appointed Director-General of the International Health Commission, and Dr. John A. Ferrell was appointed Assistant Director-General, the President and the Secretary of the Foundation serving ex-officio as Chairman and Recording Secretary respectively.

### What the Commission Has Undertaken to Do

The resolution creating the Commission assigned it to two tasks: (1) "to extend to other countries the work of eradicating hookworm disease as opportunity should offer"; and (2) "so far as practicable to follow up the treatment and cure of this disease with the establishment of agencies for the promotion of public sanitation and the spread of knowledge of scientific medicine." In keeping with this definition of purpose the Commission has directed its initial efforts to the first and more immediate task of extending to foreign countries work for the relief and control of uncinariasis or hookworm disease.

The relief and control of this one disease is an undertaking of enormous magnitude. The infection belts the globe in a zone about 66° wide, extending roughly from parallel 36° north to parellel 30° south. Practically all countries within this zone are infected. Of the 1,600,000,000 people inhabiting the globe, about 900,000,000 live in countries where the infection is prevalent.

In many countries infection is extremely prevalent. Of 548,992 rural children microscopically examined in the Southern States, 39 per cent. were found to be infected. Reports received by the Rockefeller Sanitary Commission in 1911, and summarized in its Publication No. 6, estimate: that of the population of Columbia living between sea level and 3,000 feet above, 90 per cent. are infected; that of the population of British Guiana, 50 per cent. are infected, the infection among the coolies on sugar estates being even greater; that in Dutch Guiana the infection on many plantations runs as high as 90 per cent; that in Egypt the infection of the laboring population is approximately 50 per cent.; that 50 per cent. of the Indian coolies on sugar and tea estates in Natal are infected; that on many plantations in Ceylon the infection runs as high as 90 per cent.; that there is an extremely heavy infection in some parts of India and among the coolies on many estates in Malaya and Fiji which import their labor from India; that the southern two-thirds of the Chinese Empire is involved, the infection in many parts of the Yangtse Valley running as high as 70 to 76 per cent. among the farming population.

The relief and control of the disease in a given country involves: (1) making a survey to determine the geographic distribution and the approximate degree of infection; (2) miscroscopically examining the people and curing those who are found infected; and (3) setting in operation and making effective such sanitary measures as will put a stop to soil-pollution.

The International Health Commission has not undertaken to eradicate uncinariasis in any country. The accomplishment of this result will require the operation of permanent agencies working over long periods of time. The attitude assumed by the International Health Commission toward this work is that assumed by the Rockefeller Sanitary Commission in its co-operation with the Southern States, namely: that the bringing of this disease under control in any country is a work which no outside agency working independently could do if it would, and one which no outside agency should do if it could; that if the infection is to be stamped out in any area the country in which it exists must assume the responsibility; and that the Commission may be of service in so far as it may co-operate with the govenments of foreign counties in organizing and making effective their own agencies. In this spirit the Commission has accepted the invitation of eleven foreign countries during the current year to co-operate in the relief and control of this disease. It is prepared to extend this co-operation to other countries as conditions invite.

### Preliminary Conditions and Conferences

At the first meeting of the International Health Commission, June 27, 1913, the Director-General was authorized to go in person or to send a representative to British dependencies and to Latin-American countries for the purpose of preliminary investigation and conference. Travel on these missions consumed most of the time of the Director-General during the first twelve months of the Commission's existence. Three such journeys were made: (1) to England: (2) to the British West Indies; and (3) to Egypt and British dependencies in the Far East. Dr. J. H. White, of the United States Public Health Service, represented the Commission on similar journeys to countries in Central America.

As a direct consequence of these visits and of the hospitable reception given to the Commission's offer of co-operation, plans were adopted and work begun in British Guiana, Antigua, Trinidad, St. Lucia, Grenada and Egypt. A plan of work was adopted for St. Vincent, but was deferred on account of the war. In Ceylon the Government and the Planters' Association inaugurated an experimental demonstration on a small scale, in accordance with the suggestions of Mr. Rose, the entire cost being met locally. In the Malay States the consensus of opinion favored the establishment of a commission to inquire into the relative importance of hookworm disease and malaria in accounting for the physical debility of the people, and since the period under review a special commission has been appointed to make a study of this problem. In addition to the work undertaken in the

British colonies, the Commission has responded favorably to invitations from several Central American countries, and work has been inaugurated in Panama, Nicaraugua, Costa Rica and Guatemala.

In spite of the fact that a large amount of time has necessarily been given to preliminary conferences, surveys and the work of organization, a very substantial achievement has already been made, 37,902 persons having been examined, and 19,425, persons treated in all the foreign areas, up to December 31, 1914. But more significant than the number of persons treated has been the establishment of relations of co-operation and mutual confidence between the Commission and the governments and physicians of the communities visited, and the resulting stimulation of interest on the part of the common people, whereby the principle of self-help has been steadily maintained.

In addition to carrying on its work in foreign countries, the International Health Commission has also undertaken to complete the program of the Rockefeller Sanitary Commission for the eradication of hookworm disease in the Southern States. This program did not contemplete complete eradication under the supevision of the Sanitary Commission, but aimed rather at a comprehensive demonstration in each state, first of the presence of the disease, and secondly of the method of treating and preventing it. This demonstration is now entering its final stage with the inauguration of the so-called intensive community work whereby, in a limited number of typical communities in each state, it is hoped to show convincingly the possibility of treating every infected person, and at the same time of preventing soil pollution—the only way of preventing the recurrence of the disease.

METHODS BEING PURSUED.

1. Work Under Authority and Direction of Government:—In each country where this work is in progress it is being done under the authority and direction of Govenment. This is regarded as fundamental. The International Health Commission does not undertake to eradicate uncinariasis in any country; the infection can be brought under final control only by means of permanent agencies working over long periods of time. The Commission, therefore, while co-operating with Governments in the work of immediate relief, seeks to do this in such a way as to aid in building up permanent public health agencies for the control of this disease and all other diseases.

2. Work Begins on Small Scale:—The plan of work adopted for each country makes provisions for beginning operations on a small scale. This has distinct advantages. The opening of work in each new country must be in the nature of an experiment. By beginning operations on a small scale opportunity is given, without waste of funds, to try out agencies and methods until they have become adjusted to local conditions. When the

1. Work Under Authority and Direction of Government:—In each country where this work is in progress it is being done under the authority and direction of Government. This is regarded as fundamental. The International Health Commission does not undertake to eradicate uncinariasis in any country; the infection can be brought under final control only by means of permanent agencies working over long periods of time. The Commission, therefore, while co-operating with Governments in the work of immediate relief, seeks to do this in such a way as to aid in building up permanent public health agencies for the control of this disease and all other diseases.

2. Work Begins on Small Scale.—The plan of work adopted for each country makes provisions for beginning operations on a small scale. This has distinct advantages. The opening of work in each new country must be in the nature of an experiment. By beginning operations on a small scale opportunity is given, without waste of funds, to try out agencies and methods until they have become adjusted to local conditions. When the effective working unit for these conditions has been ascertained this unit can be multiplied at will. On opening work in a new country it becomes necessary also to train a local staff of microscopists, nurses, and caretakers for the service. When it has become standardized, the servise itself is the best trainingschool for its own employes; and the training of employees goes hand in hand with the enlargement of the work.

3. Treating the Infected:—In all these countries the work is organized with a view to centering first effort on measures of relief,—that is, on treating the people who are found infected.

Systematic treatment is justifiable if only as a means of relieving suffering and inefficiency. Within the brief period during which the work has been in operation 19,425 persons have been treated. The significance of this result is to be stated not primarily in medical terms, as the relief of 19,425 people; but in education terms, as the instructing and moving to action of a much larger number of peole. For every person successfully treated becomes the effective teacher of a circle of friends and neighbors.

4. Infection Survey:—Effort is being made in each country concerned to carry out a survey to determining the geographic distribution of the infection, and to estimate the degree of infection for each infected area.

5. Preventive Measures:—The organization in each of the eleven countries is conducting a sanitary survey to determine the existing conditions responsible for the presence and spread of uncinariasis in the infected areas. In Egypt, where the absence of ground-itch has given rise to doubt as to whether the infection in transmitted chiefly through the skin or by the mouth, this survey has for its object: (1) to ascertain how the infection is transmitted among the fellahin; and (2) to locate the danger-points about the Egyptian village from which the infection is spread. The final purpose of the survey in Egypt, as in all the other countries, is to lay the basis for a system of sanitary measures designed to bring the disease under control.

6. Educating the People:—This work is essentially educational; it is teaching the people by demonstration. The field directors carry the work out among the people. They tell the story of this disease in varied graphic forms and in terms so simple that the common man, though he be illiterate, may see and understand. In the Southern States the schools and the public press were enlisted and large use was made of pamphlets, leaflets, and circular letters. These agencies are not being neglected in the foreign field; but among the natives in many of the tropical countries the story must be presented in more direct and concrete terms. Here the field directors rely more upon telling the story by word of mouth; and as they tell it they ilustrate its details by means of lantern-slides, photographs, and objects. They use typical cases as object-lessons; they point out the gross clinical symptoms in these cases (and these the people soon learn to recognize); they get specimens of the patients' stools and exhibit the eggs of the parasite under the microscope; they show the parasites that have been expelled by the treatment administered; and by means of the microscope they exhibit the living, squirming embryos that live by teeming thousands in the soil that has been befouled by an infected person, and are ready to infect any person whose bare skin they come into contact. The recovery that follows treatment and cure tells its own story, both to the patient and to his friends and neighbors. The disease thus lends itself so readily to simple demonstration that the peole—even native populations of tropical countries—easily understand its whole story. They learn to recognize the disease by its clinical picture; they have seen the parasite that causes it, and the eggs by which infection is demonstrated; and they see how the infection is spread and how it may be prevented. As a result of this educational work, the people cooperate helpfully, in both the work of treatment and that of prevention.

7. An Object Lesson in the Treatment of Disease:—The relief and control of this one disease is an object lesson in the relief and control of disease in general. This one is simple and tangible; the common man can easily understand what it is, and what it means to him as a menace to his health and to his earning power; he knows its whole story; he knows its simple treatment and its one simple preventive measure. Having seen this one disease brought under control and having had the worth of the effort brought home to him, he is prepared to give heed when spoken to about the control of diseases that are less simple and less tangible. To repeat, then, for the sake of emphasis, this whole work is essentially educational; and its best result is in securing the helpful co-operation of the people in the work of bringing this disease and all other preventable diseases under control.

---

### FIFTY FALSELY LABELED MEDICINES.

### Federal Courts Condemn Goods or Fine Many Patent Medicine Manufacturers. Fifty Patent Medicines Proceeded Against for Fraudulent Claims As to Curative Powers of Products.

More than half a hundred legal actions have been terminated successfully under the Sherley Amendment to the Food and Drugs Act, which prohibits false and fraudulent claims as to the curative or therapeutic effects of drugs or medicines. Criminal prosecutions against the manufacturers were brought in 25 cases, but in 31 instances the falsely and fraudulently labeled medicines were seized while in interstate commerce. Claims made by the manufacturers for the curative powers of these preparations ranged from tuberculosis, smallpox and diphtheria to coughs, colds and scalp diseases. A number of other criminal prosecutions and seizures are pending in various Federal Courts throughout the United States because of alleged violations of the Sherley Amendment similar to those which have already been tried. The officials charged with the enforcement of the Food and Drugs Act are of the opinion that the evils of the patent medicine business can be stopped only by the most drastic action.

It is pointed out that traffic in medicines for which false and fraudulent claims are made is not only an economic fraud of the worst kind, in that a worthless preparation that costs but a few cents is frequently sold for a dollar or more a bottle, but that health, and even life, is endangered by failure to secure the service of a physician in such serious diseases as tuberculosis, diphtheria, pneumonia and scarlet fever, until too late, because reliance may have been placed in the curative powers of some worthless preparation which is claimed to be a never-failing remedy. The deluded victim may not realize his danger until the disease has reached a stage too far advanced for even the ablest physicians to cope with it. Effective treatment depends in most cases on applying it during the early stages of the disease.

### Suggestive Name of "Family Physician" Fails to Save This Preparation.

The Houchens Medicine Company of Baltimore, Md., pleaded guilty to the charge that a preparation called "Family Physician" and shipped by them into interstate commerce was falsely and fraudulently labeled. Among the many diseases for which this medicine was recommended by the manufacturers in statements appearing on the labels and accompanying circulars were diphtheria, scarlet fever, typhoid fever, smallpox, bronchitis, neuralgia, croup and all diseases of the throat and lungs. The following quotations from the label, carton, or circular are interesting: "The Public is hereby assured that this is the Genuine and Original Family Physician. * * * For fever you need not give anything else but this Medicine, it will keep the rash out itself. * * * * For cases of Small Pox take plenty and often—Use freely. Give no hot teas, just give the medicine and what pimples are under the skin will come out, the rest will be carried off by the medicine. * * * * Also a wonderful and positive remedy for dyspepsia, keeps measles out nicely, regulates the bowels without trouble, and by purifying the blood prevents your liability to disease."

Analysis of the product, which was claimed by the manufacturer to be effective in the treatment of so many virulent and contagious diseases, as well as a variety of minor ills, showed that it was a sirup containing 19.2 per cent of non-volatile matter, 8.9 per cent. alcohol, anise, and a vegetable cathartic drug. . The Government, therefore, charged that the medicine did not contain ingredients or medicinal agents effective for the relief and cure of the diseases which it was claimed to cure. The court imposed a fine of $75.

### Remarkable Claims for Dr. H. A. Inghram's Vegetable Expectorant Nervine Pain Extractor

A plea of guilty was entered by H. A. Ingham and Co.,. .of Vergennes, Vt., to the charge that statements and claims as to curative powers of a product called "Dr. H. A. Ingham's Vegetable Expectorant Nervine Pain Extractor" were false and fraudulent. An analysis of a sample of the product by the Bureau of Chemistry showed the same to contain alcohol, 86 per cent.; opium alkaloids, camphor, capsicum, and vegetable extractive matter. The Government, therefore, alleged that the medicine did not contain ingredients or medicinal agents effective, as the labels or circulars asserted, to subdue raging fever, or to cure typhoid fever, lung fever, scarlet fever, rheumatic fever, cholera, dysentery, sunstroke, diphtheria, bleeding at the lungs, nervous exhaustation, or piles, or to prevent fits of apoplexy and epilepsy when coming on, or to heal without inflammation or suffering all wounds, sprains, or burns, or to break up a felon, or to cure congestion of the lungs, pleurisy, fits of apoplexy, chronic rheumatism, paralyzed limbs, and croup.

It was also alleged by the Government that the statements "For teething and restless children, it is not only safe and harmless, but positively beneficial; it agrees with the most tender child or feeble infant," were false and misleading in that they were of such nature as to mislead the purchasers into the belief that the article contained no harmful or poisonous ingredient. whereas, in fact it did contain morphine and other opiumalkaloids of a poisonous and deleterious nature, such as might prove harmful and deleterious to the health of tender children and feeble infants, and other persons, if consumed by them. The court fined the defendant $100.

### Seized Four Thousand Bottles of "Father John's Medicine."

Four thousand and ninety-two bottles of "Father John's Medicine" were seized in Philadelphia, Pa., it being alleged in the libel that the labels on the bottles and on the pasteboard packages containing the bottles bore statements regarding the curative effects of the medicine that were false and fraudulent. Claims were made by the manufacturers for the efficacy of the medicine in the treatment of consumption, coughs, colds, croup, asthma, bronchitis, sore throat, whooping cough, pneumonia, catarrh, rickets, and a number of other ailments. A judgment of condemnation and forfeiture was entered, and it was ordered by the court that the product be delivered to Carleton and Hovey Company, Lowell, Mass., upon payment of all the costs in the proceedings and the execution of a bond in the sum of $5,000, to insure that the goods would not be sold unless truthfully relabeled.

Jury Says "Guilty" for Misbranding "Bad-Em-Saltz."

A verdict of "guilty" was rendered against the American Laboratories, a corporation located at Philadelphia, Pa., for shipping into interstate commerce a product called "Bad-Em-Salz" which it was alleged was falsely and fraudulently labeled. An analysis of a sample of the product showed that it consisted of common salt, Glauber salt, baking soda, and a small amount of tartaric acid. It was claimed by the manufacturers that this preparation reproduced the medicinal properties of the great European springs famous for centuries for the cure of diseases of the stomach, intestines, liver, kidneys, or bladder, and that it represented the medicinal agents obtained by the evaporating of the water from famous European springs. The Government alleged among other things that these claims were false and misleading. It was also alleged that the statements in the circular indicating that the preparation contained ingredients or medicinal agents effective for dissolving gall stones, for the prevention of gastritis, for curing diabetes, for preventing or checking chronic inflammation of the kidneys, and for relieving catarrh of the bladder, were false and fraudulent. A fine of $100 was imposed by the court.

Long List of Other Misbranded Medicines.

The following list includes other preparations against which the Government's charge that they were falsely or fraudulently labeled was sustained by the Federal courts. Statements were made on the labels of, or on the circulars accompanying, the preparations intended to make the purchaser believe that the medicines were effective cures for a great variety of diseases for which they were recommended by the manufacturers or promoters. The main allegations of the Government were upheld by the courts and judgment accordingly entered in connection with each of the following preparations:

Radam's Microbe Killer, Hilton's Specific, Smith's Agricultural Liniment, Dr. Sullivan's Sure Solvent, Russell's White Drops, Stramoline, Wild Cherry Pepsin, Moreau's Wine of Anise, Dr. Herman Koch's Brand Phosphates, Celery and Gin Compound; Swissco Hair and Scalp Remedy, Cod Liver Oil with Syrup of Tar, Dr. Mozley's Lemon Elixir, Sa-Yo Mint Jujubes, Gray's Glycerine Tonic Compound, Dr. Martel's Female Pills, Quickstep—Frye's Remedy, Seawright's Magnesian Lithia Water, Hill's Aromatic Ext. Cod Liver Oil (Hollander-Koshland Co), Black's Pulmonic Syrup, Tetterine, Laxative Quinine Tablets, Mrs. Joe Person's Remedy, Maignen Antiseptic Powder, Cranitonic Scalp Food-Hair Food, Dr. David Kennedy's Cal-Cura Solvent, Schenck's Pulmonic Syrup, Keller's Flaxseedine, Tutt's Pills, Universal Rheumatic Remedy, Green Mountain Oil, Weber's Genuine Alpine Herb Tea, Montague's Liniment, Coe's Cough Balsam, White Stone Lithia Water, Kalamazoo Celery & Sarsaparilla Compound, Quality Damiana Compound, Dennis Eucalpytus Ointment, Cassidy's 4X The Great Blood Purifier, Porter's Antiseptic Healing Oil, Ballard's Horehound Syrup Compound, Dr. Shoop's Night Cure, Dr. Shoop's Cough Remedy, Dr. Shoop's Restorative, Rheumacide, Rice's Mothers' Joy Salve, Milam, Old Jim Fields Phosphate Dill and Gin, Stuart's Buchu and Juniper Compound, Ozomulsion, Jones' Break Up, Carswell's Liver Aid, Dr. Shoop's Twenty Minute Croup Remedy, Rogers' Consumption Cure and Cough Lozenges, Rogers' Inhalent.

